# Prostate zonal impact of 5α‐reductase inhibitors on multiparametric MRI characteristics and detection of prostate cancer

**DOI:** 10.1111/bju.70067

**Published:** 2025-11-05

**Authors:** Arighno Das, Marcelo Bigarella, Krista Brackman, Glenn O. Allen, Soroush Rais‐Bahrami, Kayla Bahr, Emily Schmitt, Andrew M. Fang, Hunter Boudreau, Christopher P. Filson, Melina Pectasides, Giuseppe V. Toia, David F. Jarrard

**Affiliations:** ^1^ Department of Urology, School of Medicine and Public Health University of Wisconsin Madison WI USA; ^2^ Department of Radiology and Medical Physics University of Wisconsin Madison WI USA; ^3^ Carbone Comprehensive Cancer Center University of Wisconsin Madison WI USA; ^4^ Department of Urology University of Alabama at Birmingham Heersink School of Medicine Birmingham AL USA; ^5^ Department of Radiology University of Alabama at Birmingham Heersink School of Medicine Birmingham AL USA; ^6^ O'Neal Comprehensive Cancer Center University of Alabama at Birmingham Heersink School of Medicine Birmingham AL USA; ^7^ Department of Urology Emory University School of Medicine Atlanta GA USA; ^8^ Winship Cancer Institute Emory Healthcare Atlanta GA USA; ^9^ Department of Radiology Emory University School of Medicine Atlanta GA USA; ^10^ Atlanta VA Medical Center Decatur GA USA

**Keywords:** 5α‐reductase inhibitor, apparent diffusion coefficient, multiparametric MRI, prostate biopsy, prostate cancer

## Abstract

**Objectives:**

To assess the impact of 5α‐reductase inhibitors (5‐ARIs) on multiparametric magnetic resonance imaging (mpMRI) features of Prostate Imaging‐Reporting and Data System (PI‐RADS) lesions and their influence on the detection of clinically significant prostate cancer (csPCa), with a focus on differences between prostate zones.

**Patients and Methods:**

We retrospectively reviewed data from 1108 PI‐RADS version 2 score 3–5 lesions in 718 patients across a multi‐institutional cohort, all of whom underwent magnetic resonance imaging (MRI)‐targeted biopsy. A subset of 66 lesions from patients receiving 5‐ARI therapy was matched to lesions from untreated patients and independently reviewed by an experienced radiologist in a blinded fashion. Apparent diffusion coefficient (ADC) values were quantified for each lesion and for peripheral and transition zones.

**Results:**

Among the 1108 lesions, 90 (8%) were in patients on 5‐ARI therapy for ≥3 months prior to mpMRI. Multivariable analysis demonstrated that 5‐ARI use was associated with significantly reduced odds of detecting csPCa on targeted biopsy (odds ratio [OR] 0.32, 95% confidence interval [CI] 0.18–0.57). Stratified analysis revealed a pronounced reduction in csPCa detection in peripheral zone lesions (OR 0.20, 95% CI 0.05–0.68), but not in transition zone lesions (OR 0.38, 95% CI 0.11–1.18). Blinded radiological review showed higher mean ADC values in peripheral zone lesions among 5‐ARI users (869 vs 765 mm^2^/s; *P* = 0.04) and lower lesion conspicuity (*P* = 0.027). There were no significant imaging differences in the transition zone.

**Conclusions:**

Treatment with 5‐ARIs is associated with decreased detection of csPCa on MRI‐targeted biopsy, especially for peripheral zone lesions. These effects may be attributable to increased ADC values and reduced lesion conspicuity, suggesting a potential increase in false‐positive mpMRI findings in patients receiving 5‐ARIs.

**Abbreviations**
ADC: apparent diffusion coefficient
5‐ARI: 5α‐reductase inhibitor
GG: Grade Group
mpMRI: multiparametric MRI
OR: odds ratio
(cs)PCa: (clinically significant) prostate cancer
PCPT: Prostate Cancer Prevention Trial
PI‐RADS(v2.1): Prostate Imaging‐Reporting and Data System (version 2.1)
PZ: peripheral zone
REDUCE: Reduction by Dutasteride of Prostate Cancer Events Trial
TZ: transition zone

## Introduction

Multiparametric MRI (mpMRI), along with mpMRI‐TRUS fusion‐targeted biopsy, has become central to the detection and risk stratification of prostate cancer (PCa). Targeted biopsies improve the detection of clinically significant PCa (csPCa), defined as Grade Group (GG) ≥2 [1, 2]. However, the prostate's distinct zonal anatomy—comprising the peripheral zone (PZ) and transition zone (TZ)—poses diagnostic challenges. Tissue heterogeneity, particularly in the TZ, can obscure the distinction between csPCa and BPH on mpMRI [3, 4].

The 5α‐reductase inhibitors (5‐ARIs) specifically inhibit 5α‐reductase (α1c) enzyme required for conversion of testosterone to dihydrotestosterone. These medications are widely utilised for the management of BPH and LUTS [5]. Landmark trials including the Prostate Cancer Prevention Trial (PCPT) and Reduction by Dutasteride of Prostate Cancer Events Trial (REDUCE) demonstrated a reduction in the cumulative PCa incidence following treatment with 5‐ARIs [6, 7]. However, an increased incidence of high‐grade tumours (GG 4–5) was reported in a subset of patients on 5‐ARIs [8]. Long‐term follow‐up of the PCPT trial demonstrated no significant differences in the overall survival or cancer‐specific survival between treated and untreated groups [9].

Importantly, both the PCPT and REDUCE predated the widespread use of mpMRI. As such, the impact of 5‐ARI therapy on mpMRI lesion characteristics and the diagnostic performance of fusion biopsy remains poorly defined. Preliminary studies suggest that 5‐ARI use may alter apparent diffusion coefficient (ADC) values, but the clinical implications of these imaging changes are unclear [10, 11, 12, 13]. Moreover, prior analyses often include both template and targeted biopsies, potentially confounding the relationship between 5‐ARI use and mpMRI‐targeted biopsy performance [11, 14, 15].

Given the widespread use of 5‐ARIs and the growing reliance on mpMRI for biopsy decision‐making, we aimed to characterise the influence of 5‐ARI use on csPCa detection via targeted biopsy, with particular attention to zonal anatomy. We conducted a two‐part study: (i) a retrospective, multi‐institutional analysis comparing csPCa detection rates by zone in patients on 5‐ARIs vs untreated patients; and (ii) a matched cohort analysis assessing the impact of 5‐ARIs on mpMRI lesion characteristics.

## Patients and Methods

### Patient Population

Clinical, imaging, and pathological data were collected from consecutive patients undergoing mpMRI for PCa evaluation at two tertiary referral centres between 2015 and 2019, under Institutional Review Board‐approved protocols. Patients with Prostate Imaging‐Reporting and Data System version 2.1 (PI‐RADSv2.1) lesions scored >2 were included. All imaging was reviewed and confirmed by fellowship‐trained radiologists with experience in reading and reporting prostate MRIs. All patients underwent MRI–TRUS fusion‐targeted biopsy of an index PI‐RADSv2.1 lesion. The use of additional template biopsies was at the discretion of the treating physician. MRI protocols are listed in Table S1.

Collected clinical variables included age, race, history of prior biopsy, and PSA level at the time of biopsy. Patients in the 5‐ARI cohort had been receiving 5 mg finasteride or 0.5 mg dutasteride daily for a minimum of 3 months prior to mpMRI, based on data indicating that prostate volume and PSA stabilise after this period [16, 17]. For patients on 5‐ARI, PSA values were doubled to account for the suppressive effect of the medication [18].

### Biopsy Protocol and Histopathology

Targeted biopsies were taken in all patients. The prostate and PI‐RADSv2.1 score 3–5 lesions were manually contoured (DynaCAD, Philips Healthcare, Cambridge, MA, USA) by fellowship‐trained abdominal radiologists or Society of Urologic Oncology fellowship‐trained urologic oncologists based on institutional protocols and imported to ultrasound (bk3000, BK Medical, Burlington, MA, USA) with image‐fusion software (UroNav, Philips Healthcare) at all centres. Tissue cores obtained from the targeted and template biopsies were labelled and fixed separately.

### Radiology Analysis

A subset of mpMRI lesions from patients on 5‐ARIs at the time of imaging were matched in a 1:1 fashion with lesions from untreated patients based on age, corrected PSA, prostate volume, and PI‐RADSv2.1 score. Matching methodology is described below. After matching, an experienced fellowship‐trained radiologist (G.V.T.), blinded to 5‐ARI status, evaluated all lesions.

For each case, the ADC was measured for the lesion, the non‐tumorous PZ, and the TZ. Tumour conspicuity was defined as the ratio of ADC of the surrounding normal zone (PZ or TZ) to that of the lesion. Conspicuity for PZ lesions was calculated as PZ ADC/lesion ADC, following the method described by Giganti et al. [19]. A similar formula was used for TZ lesions.

### Statistical Analysis

Statistical analysis was conducted using RStudio (Boston, MA, USA). Group comparisons for baseline variables were performed using Student's *t*‐test, Mann–Whitney *U* test, or chi‐square test as appropriate. Multivariable logistic regression was used to assess associations between 5‐ARI use and csPCa detection, with final pathology from targeted biopsy as the dependent variable. Reporting guidelines for STrengthening the Reporting of OBservational studies in Epidemiology (STROBE) were followed.

Lesion matching was performed using the ‘MatchIt’ package in R, which incorporates functions from the ‘Matching’ package [20, 21]. Matching techniques tested included coarsened exact matching, nearest neighbour matching (with and without replacement), and genetic matching. Genetic matching was selected for its performance, as it achieved standardised mean differences <0.1 and variance ratios near 1. Matching variables included age, corrected PSA, prostate volume, and PI‐RADSv2.1 score [20].

To assess the effect of 5‐ARI use on lesion characteristics and biopsy outcomes post‐matching, logistic regression models were constructed. Marginal effects and standard errors were estimated using the ‘glm’ function from base R and ‘avg. comparisons’ from the ‘marginal effects’ package [22].

## Results

### Patient Cohort and Baseline Characteristics

A total of 1108 PI‐RADSv2.1 3–5 lesions from 718 mpMRI scans were identified. Among these, 60 patients (8.3%) were on 5‐ARI therapy for ≥3 months at the time of imaging. The median (interquartile range) duration of 5‐ARI use was 41 (7.3–73.5) months. Patients on 5‐ARI were older and had higher corrected PSA values compared to untreated patients; however, the number of lesions per patient did not differ significantly. Full clinical characteristics are summarised in Table 1.

**Table 1:** Baseline characteristics of patients included in the multi‐institutional cohort.

Characteristic, median (IQR)	5‐ARI use, *N* = 60	No 5‐ARI use, *N* = 658	*P*
Age, years	70 (66–73)	66 (61–70)	<0.001
Corrected PSA level, ng/mL	16 (11–25)	8 (6–12)	<0.001
Prostate volume, mL	56 (37–78)	48 (36–68)	0.12
Number of PI‐RADSv2.1 3–5 lesions	1 (1–2)	1 (1–2)	0.8

IQR, interquartile range. Wilcoxon rank sum test. Of the 1108 lesions, 90 (8.1%) were from patients on 5‐ARIs. PI‐RADSv2.1 lesion characteristics are detailed in Table 2. A greater proportion of PI‐RADSv2.1 4 and 5 lesions were observed among patients on 5‐ARIs (80% vs 64%, *P* = 0.01, chi‐square test). There were no significant differences in lesion size or active surveillance status between the two groups.

### Detection of csPCa

**Fig. 1:**
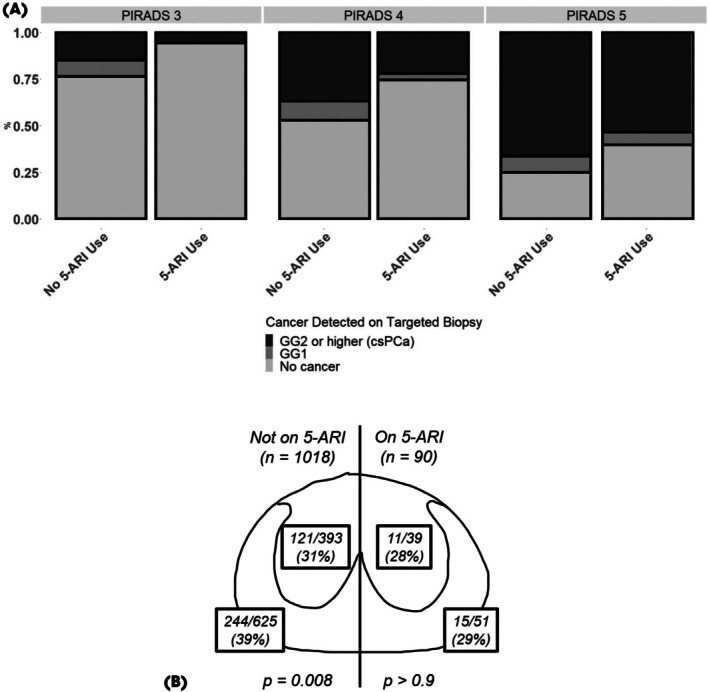
(**A**) Differences in detection of prostate cancer by PI‐RADSv2.1 score and 5‐ARI status within multicentre cohort. Of 1108 lesions in a multicentre cohort, 90 were on 5‐ARIs at the time of imaging and biopsy. For all PI‐RADSv2.1 scoring, a lower percentage of csPCa was found in the directed lesion biopsies of patients on 5‐ARIs. (**B**) TZ and PZ csPCa detection in patients on 5‐ARIs vs no 5‐ARI use.

The primary outcome was detection of csPCa (GG 2–5) on targeted biopsy. Detection rates stratified by PI‐RADSv2.1 score and 5‐ARI status are shown in Fig. 1A. In multivariable analysis adjusted for PI‐RADSv2.1 score, 5‐ARI use was associated with significantly lower odds of csPCa detection (odds ratio [OR] 0.53, 95% CI 0.31–0.87; *P* = 0.014). This association remained significant after adjusting for age, corrected PSA, prostate volume, PI‐RADSv2.1 score, and active surveillance status (OR 0.32, 95% CI 0.18–0.57; *P* < 0.001). Notably, despite a higher frequency of PI‐RADSv2.1 4 and 5 lesions in the 5‐ARI group, csPCa detection rates were lower.

### Zonal Analysis

We assessed whether the effect of 5‐ARI use varied by zonal anatomy. The distribution of lesion location (PZ vs TZ) was similar between groups (61% vs 57% in the PZ; *P* = 0.1, Table 2). Among patients not on 5‐ARI, csPCa was more frequently detected in PZ lesions compared to TZ lesions (39% vs 31%, *P* = 0.008, Fig. 1B). In contrast, among patients on 5‐ARI, csPCa detection rates did not differ between PZ and TZ (29% vs 28%, *P* = 0.9).

**Table 2:** Baseline clinical characteristics of PI‐RADSv2.1 3–5 lesions identified in the multi‐institutional cohort.

Characteristic	5‐ARI use, *N* = 90	No 5‐ARI use, *N* = 1018	*P*
Age, years, median (IQR)	70 (66–73)	65 (61–70)	**<0.001**
PSA level, ng/mL, median (IQR)	8 (5–13)	8 (6–12)	0.6
Corrected PSA level, ng/mL, median (IQR)	16 (10–26)	8 (6–12)	**<0.001**
Prostate volume, mL, median (IQR)	60 (37–83)	49 (36–70)	**0.016**
PI‐RADSv2.1 score, *n* (%)			
3	18 (20)	365 (36)	**0.01**
4	44 (49)	407 (40)	
5	28 (31)	246 (24)	
Maximum MRI lesion dimension, cm, median (IQR)	1.40 (1.00–1.90)	1.30 (1.00–1.70)	0.11
Active surveillance, *n* (%)	15 (17)	250 (25)	0.093
PZ lesion, *n* (%)	51 (57)	625 (61)	0.4

IQR, interquartile range. Wilcoxon rank sum test; Pearson's chi‐squared test. Bold values statistically significant at *P* < 0.05.

### Matched Cohort Analysis

To address the imbalance between groups, the 90 lesions from patients on 5‐ARI were matched 1:1 with lesions from untreated patients. Baseline characteristics for the matched cohort are shown in Table S2. In logistic regression of the matched dataset, 5‐ARI use remained associated with significantly reduced odds of csPCa detection (marginal OR 0.41, 95% CI 0.24–0.68; *P* = 0.03).

We then assessed zonal differences within this matched cohort (*N* = 180 lesions: 104 PZ, 76 TZ). Consistent with the full cohort, 5‐ARI use was associated with significantly reduced odds of csPCa detection in PZ lesions (OR 0.20, 95% CI 0.05–0.68; *P* = 0.016), but not in TZ lesions (OR 0.38, 95% CI 0.11–1.18; *P* = 0.11).

### The ADC and Conspicuity Analysis

To investigate potential imaging‐based mechanisms underlying reduced csPCa detection, we conducted a blinded radiological analysis. A total of 66 lesions from patients on 5‐ARI were matched to 66 lesions from untreated patients (*N* = 132); ADC values could not be calculated for three lesions due to incomplete imaging (two due to artefact from rectal air and one due to artefact from artificial hip), leaving 129 evaluable lesions (69 PZ, 60 TZ).

Among PZ lesions, the average lesion ADC was significantly higher in the 5‐ARI group (869 vs 765 mm^2^/s; *P* = 0.04, Table 3). No difference was found in the ADC of adjacent non‐tumour peripheral tissue. Lesion conspicuity—defined as the ratio of surrounding zone ADC to lesion ADC—was significantly lower in the 5‐ARI group (1.59 vs 1.78; *P* = 0.03). In the TZ, there was a non‐significant trend toward lower lesion ADC values in the 5‐ARI group (780 vs 811 mm^2^/s; *P* = 0.13). There were no significant differences in non‐tumour tissue ADC or lesion conspicuity between the treated and untreated groups (Table 3). Representative images are shown in Fig. S1.

**Table 3:** (Top) Imaging characteristics of PZ lesions in matched patients on 5‐ARIs vs no 5‐ARI use. (Bottom) Imaging characteristics of TZ lesions in matched patients on 5‐ARIs vs no 5‐ARI use.

Characteristics PZ lesions, median (IQR)	5‐ARI use, *N* = 37	No 5‐ARI use, *N* = 32	*P*
Average PZ lesion ADC, mm^2^/s	869 (706–978)	765 (646–844)	0.04
PZ ADC, mm^2^/s	1328 (1208–1472)	1376 (1296–1512)	0.2
PZ lesion conspicuity	1.59 (1.37–1.87)	1.78 (1.63–2.12)	0.03

Characteristics TZ lesions, median (IQR)	5‐ARI use, *N* = 28	No 5‐ARI use, *N* = 32	*P*
Average TZ lesion ADC, mm^2^/s	780 (673–858)	811 (711–966)	0.13
TZ ADC, mm^2^/s	1066 (1050–1152)	1182 (1067–1228)	0.07
TZ lesion conspicuity	1.36 (1.25–1.68)	1.38 (1.23–1.59)	0.7

IQR, interquartile range. Wilcoxon rank sum test; Wilcoxon rank sum exact test.

## Discussion

Multiparametric MRI has become a cornerstone in the diagnosis and risk stratification of prostate cancer, enhancing the detection of csPCa through targeted biopsy techniques [23, 24]. Prior studies have demonstrated that use of 5‐ARIs can result in decreased vascularity of the prostate, reduce prostate cancer tumour burden, and drive distinct molecular changes to prostatic luminal cells [25, 26, 27]. The implications of these histological alterations on mpMRI performance remain poorly understood. Our study provides compelling evidence that 5‐ARI use is associated with distinct imaging changes specifically, increased ADC and reduced lesion conspicuity in PZ PI‐RADS lesions. Importantly, these changes correlated with a significant reduction in the detection of csPCa on targeted biopsy, particularly in the PZ. Lesion size remained comparable between treated and untreated patients, suggesting that imaging changes, not lesion volume, drive the difference in detection rates.

Using a large multi‐institutional cohort of 1108 PI‐RADSv2.1 3–5 lesions from 718 patients, we demonstrated that 5‐ARI use was independently associated with lower odds of csPCa detection, even after adjusting for potential confounders such as age, corrected PSA, prostate volume, PI‐RADSv2.1 score, and active surveillance status. This relationship persisted in a matched cohort analysis (Fig. 1), supporting the robustness of our findings.

Previous studies have yielded mixed results regarding the impact of 5‐ARI on detection of csPCa on targeted biopsies of mpMRI lesions [11, 14, 15]. In part those studies focused solely on the highest grade ‘index’ lesion and included csPCa detected via both targeted and template biopsies, which dilutes the specific diagnostic value of mpMRI. In contrast, our analysis considered all PI‐RADSv2.1 3–5 lesions individually and focused strictly on csPCa detected from targeted biopsies. This approach allowed for a more granular evaluation of how 5‐ARI exposure may alter the diagnostic accuracy of mpMRI.

Our zonal analysis revealed that the reduction in csPCa detection was specific to PZ lesions, with no significant change observed in TZ lesions. To investigate the mechanism behind this zonal difference, we conducted a blinded radiological analysis. We found that 5‐ARI use significantly increased lesion ADC and reduced conspicuity in PZ lesions, while no such effect was observed in the TZ. These imaging changes likely reflect underlying histopathological alterations, such as decreased cellular density or glandular atrophy, and may impair lesion visibility on diffusion‐weighted imaging.

This finding is consistent with previous evidence that ADC values increase when a cytotoxic treatment, such as chemotherapy, is applied due to an increase in water diffusion [28]. Histological changes including apoptosis and necrosis result in an increase in ADC values in PCa when exposed to androgen‐deprivation therapy [29, 30]. Interestingly, we did not see a change in the ADC or conspicuity of the TZ. The lack of change in the TZ may be due to greater baseline heterogeneity or differing microenvironmental responses to 5‐ARI exposure.

Our findings align with results from the MAPPED trial (European Clinical Trials register; EudraCT 2009‐102405‐18), a prospective randomised study that demonstrated increased ADC and reduced lesion conspicuity in PZ lesions for patients on active surveillance following dutasteride (*n* = 18 per arm) [10, 19]. Significant reductions in tumour and prostate volume were seen at 3 months (34% and 15%, respectively) with dutasteride. While our study was retrospective, it confirms these imaging changes in a larger and more diverse real‐world cohort and extends them to demonstrate a clinically meaningful reduction in csPCa detection.

Although ADC quantification is not routinely performed in clinical practice, our findings and those of the MAPPED trial suggest that 5‐ARI use should be considered during mpMRI interpretation, especially for PZ lesions. Future versions of PI‐RADS may benefit from incorporating 5‐ARI exposure as a contextual variable, potentially with adjusted ADC thresholds or altered interpretive criteria in treated patients.

This study has limitations inherent to its retrospective design, including potential selection biases. However, the use of a multi‐institutional cohort, detailed clinical and imaging data, and rigorous matching and statistical adjustment strengthen the validity of our conclusions. Patients on 5‐ARIs were older and had higher PSA values, but matching and multivariable analyses accounted for these differences. Across all analytical methods, the reduction in csPCa detection among PZ lesions remained consistent.

## Conclusion

The use of 5‐ARIs is associated with distinct changes in the mpMRI appearance of PZ lesions, including increased ADC and reduced lesion conspicuity, which correspond with a significant reduction in the odds of detecting csPCa on targeted biopsy. These findings suggest that 5‐ARI exposure may impact mpMRI interpretation and biopsy decision‐making, particularly for PZ lesions. Integration of 5‐ARI use into future iterations of PI‐RADS and mpMRI interpretation protocols may enhance diagnostic accuracy in this growing patient population.

## Disclosure of Interests

The authors have no conflicts of interest to disclose.

## Supporting information


**Fig. S1.** (A) Representative images from two patients: one on 5‐ARI (Patient #2) and one not on 5‐ARI (Patient #376). Both patients had similar risk of csPCa diagnosis (similar age, similar PSA, similar PI‐RADSv2.1 score, both PZ lesions). However, the lesion ADC and conspicuity was noticeably higher in Patient #2. Targeted biopsies of the lesion in Patient #2 were benign compared to those from Patient #376. (B) Representative images form two patients: one on 5‐ARI (Patient #210) and one note on 5‐ARI (Patient #355). Both patients had similar risk of csPCa diagnosis (similar age, similar PSA, similar PI‐RADSv2.1 score, both TZ lesions. Minimal differences in lesion ADC or conspicuity were seen. Biopsy results from both targeted biopsies returned with multiple cores of GG 3 disease.

**Table S1.** Prostate MRI of the prostate without and with contrast protocol from the three study sites, University of Alabama‐Birmingham, University of Wisconsin‐Madison, and Emory University.

**Table S2.** Baseline characteristics between two matched patient cohorts using a genetic matching algorithm.
