# Supplementary material for: Prostate zonal impact of 5α‐reductase inhibitors on multiparametric MRI characteristics and detection of prostate cancer
Source: BJU Int. 2025 Nov 5;137(2):332–8. doi: 10.1111/bju.70067 (PMC12789846; doi:10.1111/bju.70067)
Supplement: Supplementary file 1 — Fig. S1. (A) Representative images from two patients: one on 5‐ARI (Patient #2) and one not on 5‐ARI (Patient #376). Both patients had similar risk of csPCa diagnosis (similar age, similar PSA, similar PI‐RADSv2.1 score, both PZ lesions). However, the lesion ADC and conspicuity was noticeably higher in Patient #2. Targeted biopsies of the lesion in Patient #2 were benign compared to those from Patient #376. (B) Representative images form two patients: one on 5‐ARI (Patient #210) and one note on 5‐ARI (Patient #355). Both patients had similar risk of csPCa diagnosis (similar age, similar PSA, similar PI‐RADSv2.1 score, both TZ lesions. Minimal differences in lesion ADC or conspicuity were seen. Biopsy results from both targeted biopsies returned with multiple cores of GG 3 disease. [file BJU-137-332-s001.docx]

**
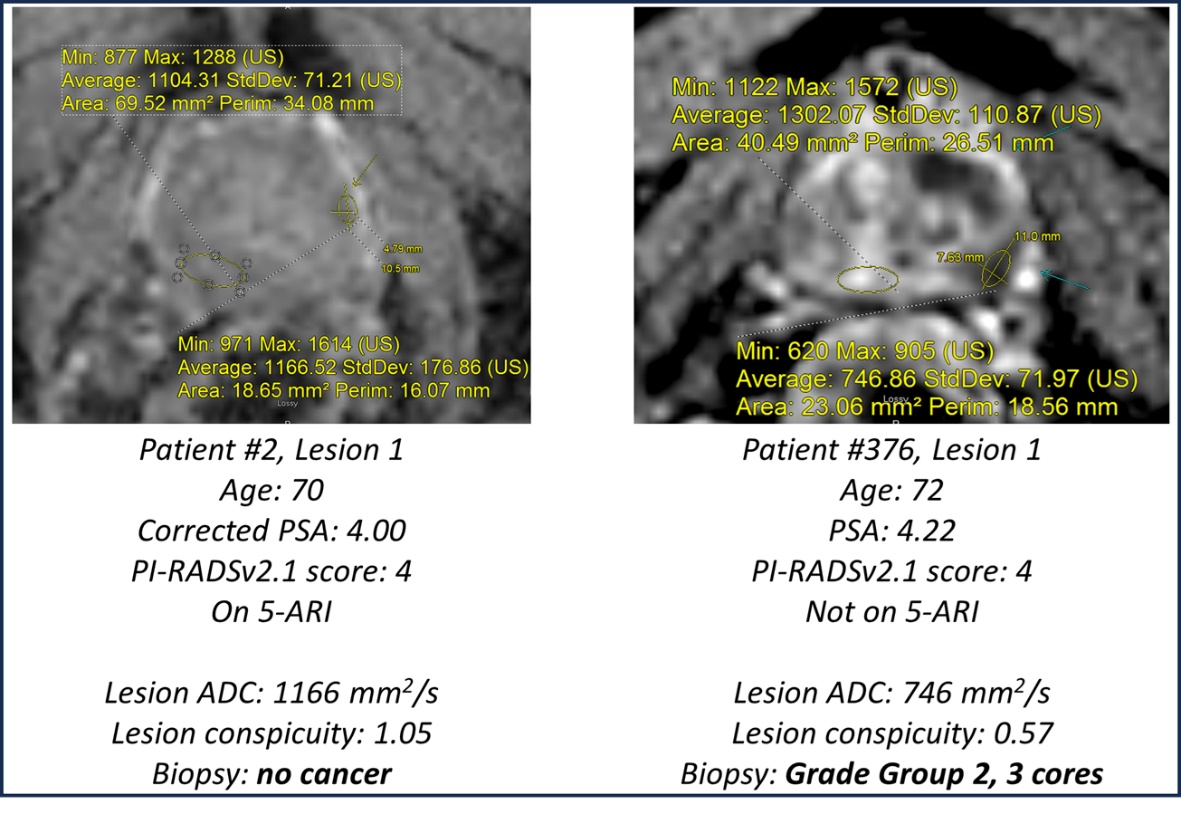
A**


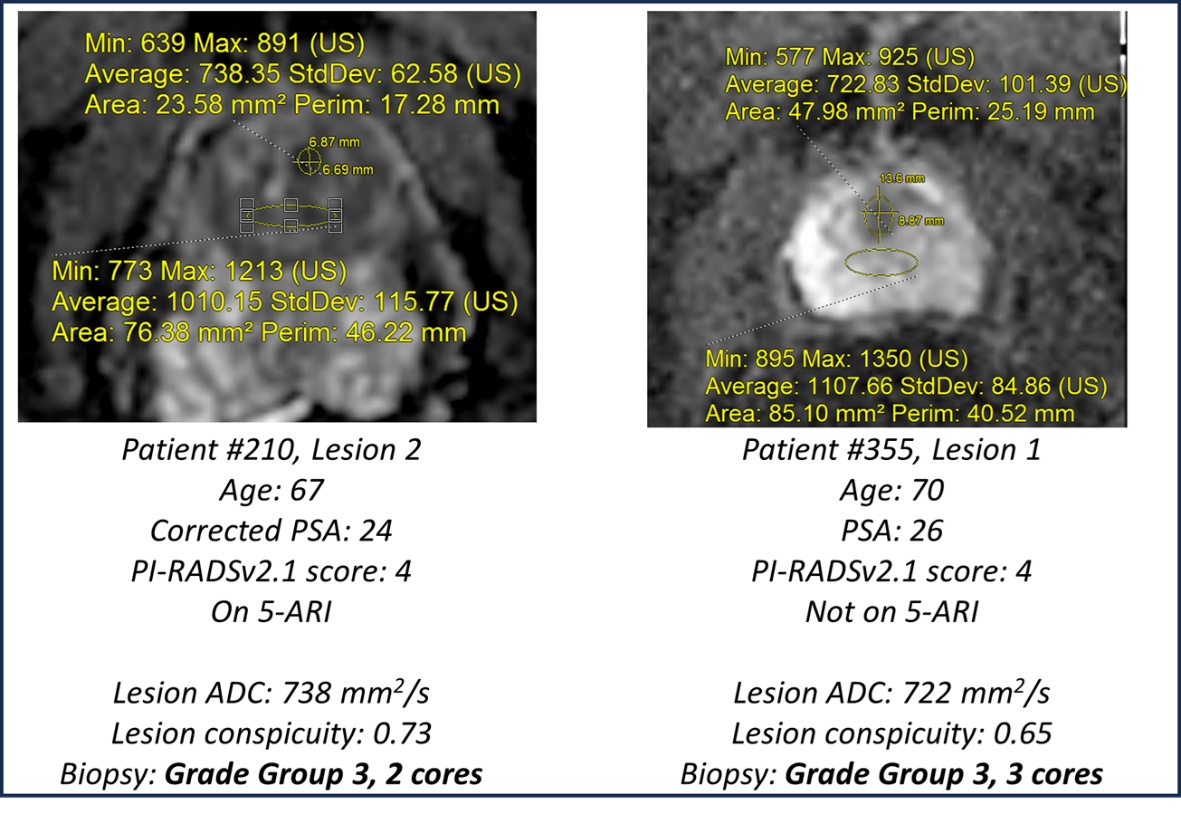


**B**

***Supplemental Figure 1: A)*** *Representative images from two patients: one on 5-ARI (Patient #2) and one not on 5-ARI (Patient #376). Both patients had similar risk of csPCa diagnosis (similar age, similar PSA, similar PI-RADSv2.1 score, both peripheral zone lesions). However, the lesion ADC and conspicuity was noticeably higher in Patient #2. Targeted biopsies of the lesion in Patient #2 were benign compared to those from Patient #376.* ***B)*** *Representative images form two patients: one on 5-ARI (Patient #210) and one note on 5-ARI (Patient #355). Both patients had similar risk of csPCa diagnosis (similar age, similar PSA, similar PI-RADSv2.1 score, both transition zone lesions. Minimal differences in lesion ADC or conspicuity were seen. Biopsy results from both targeted biopsies returned with multiple cores of Grade Group 3 disease.*
