# Supplementary material for: Prostate zonal impact of 5α‐reductase inhibitors on multiparametric MRI characteristics and detection of prostate cancer
Source: BJU Int. 2025 Nov 5;137(2):332–8. doi: 10.1111/bju.70067 (PMC12789846; doi:10.1111/bju.70067)
Supplement: Supplementary file 2 — Table S1. Prostate MRI of the prostate without and with contrast protocol from the three study sites, University of Alabama‐Birmingham, University of Wisconsin‐Madison, and Emory University. [file BJU-137-332-s003.docx]

| *University of Alabama-Birmingham* |  |  |  |
| --- | --- | --- | --- |
| **Parameters** | **T2W** | **DWI** | **DCE** |
| Field of view (mm) | (180-200) x (180-200) | (180-200) x (180-200) | (200-260) x (200-260) |
| Acquisition matrix | (320-360) x (198-240) | (64-104) x (64-104) | (128-192) x (98-154) |
| Repetition time (ms) | 3000-8000 | 3894-4238 | 3.4-5.08 |
| Echo time (ms) | 10-130 | 55-90 | 1.2-1.94 |
| Flip angle (degrees) | 90-160 | 90 | 10-25 |
| Section thickness (mm) | 3 | 3-4 | 2-4 |
| Image reconstruction matrix (pixels) | (0.5-0.6) x (0.5-0.9) x 3 | (0.87-3.1) x (0.87-3.1) x (3-4) | (1.16-1.6) x (1.16-1.6) x (2-4) |
| Time for acquisition (mins) | 3-8 | 5-10 | 5-7 |

T2W=T2 weighted; DWI=diffusion weighted imaging; DCE=dynamic contrast enhancement.

| *University of Wisconsin-Madison* |  |  |  |
| --- | --- | --- | --- |
| **Parameters** | **T2W** | **DWI FOCUS** | **DCE** |
| Field of view (mm) | 260 x 220 | 240 x 12 | 280 x 280 |
| Acquisition matrix | 384 x 256 | 120 x 60 | 224 x 200 |
| Repetition time (ms) | 4050 | 4500 | 4-7 |
| Echo time (ms) | 103 | Minimum | Minimum |
| Flip angle (degrees) | 111 | 90 | 15 |
| Section thickness (mm) | 2.4 | 4.8 | 1.3 |
| Image reconstruction matrix (pixels) | 0.7 x 1 x 2.4 | 2 x 2 x 4.8 | 1.2 x 1.4 x 2.6 |
| Time for acquisition (mins) | 4-7 | 4-8 | 4-5 |

T2W=T2 weighted; DWI=diffusion weighted imaging; DCE=dynamic contrast enhancement.

| *Emory University* |  |  |  |
| --- | --- | --- | --- |
| **Parameters** | **T2W** | **DWI** | **DCE** |
| Field of view (mm) | 200 x 200 | (200-220) x (200-220) | 300 x 300 |
| Acquisition matrix | 320 x (256-320) | (114-130) x (114-130) | 256 x 179 |
| Repetition time (ms) | 3000-7500 | 4000-5200 | 4.24 |
| Echo time (ms) | 101 | 58-63 | 1.35, 2.70 |
| Flip angle (degrees) | 140-160 | 90 | 9 |
| Section thickness (mm) | 3-3.5 | 3-3.5 | 3-3.5 |
| Image reconstruction matrix (pixels) | .63 x (.63-0.78) x (3-3.5) | (1.67-1.75) x (1.67-1.75) x (3-3.5) | 1.17 x 1.67 x (3-3.5) |
| Time for acquisition (mins) | 4:39-5:32 | 4:54-5:38 | 2:30-3:00 |

T2W=T2 weighted; DWI=diffusion weighted imaging; DCE=dynamic contrast enhancement.

**Supplementary Table 1** Prostate magnetic resonance image (MRI) of the prostate without and with contrast protocol from the three study sites, University of Alabama-Birmingham, University of Wisconsin-Madison and Emory University.
