# Supplementary material for: Prostate zonal impact of 5α‐reductase inhibitors on multiparametric MRI characteristics and detection of prostate cancer
Source: BJU Int. 2025 Nov 5;137(2):332–8. doi: 10.1111/bju.70067 (PMC12789846; doi:10.1111/bju.70067)
Supplement: Supplementary file 3 — Table S2. Baseline characteristics between two matched patient cohorts using a genetic matching algorithm. [file BJU-137-332-s002.docx]

**Supplemental Data**

| **Characteristic** | **5-ARI Use**  **N = 90** | **No 5-ARI Use**  **N = 90** | **p-value** |
| --- | --- | --- | --- |
| Age | 69.6 (65.6, 72.7) | 69.0 (66.4, 71.7) | 0.7 |
| Corrected PSA (ng/mL) | 16 (10, 26) | 16 (10, 23) | 0.6 |
| Prostate Volume (cc) | 60 (37, 83) | 57 (41, 80) | 0.7 |
| PI-RADS Score |  |  | >0.9 |
| 3 | 18 (20%) | 19 (21%) |  |
| 4 | 44 (49%) | 43 (48%) |  |
| 5 | 28 (31%) | 28 (31%) |  |
| Max MRI Lesion Dimension (cm) | 1.40 (1.00, 1.90) | 1.40 (1.10, 1.90) | 0.8 |
| Active Surveillance | 15 (17%) | 14 (16%) | 0.8 |
| Peripheral Zone Lesion | 51 (57%) | 53 (59%) | 0.8 |
| Median (IQR); n (%) | | | |
| Wilcoxon rank sum test; Pearson's Chi-squared test | | | |

***Supplemental Table 2****: Baseline characteristics between two matched patient cohorts using a genetic matching algorithm.*
